# Relative performance of indoor vector control interventions in the Ifakara and the West African experimental huts

**DOI:** 10.1186/s13071-017-2365-4

**Published:** 2017-09-19

**Authors:** Welbeck A. Oumbouke, Augustin Fongnikin, Koffi B. Soukou, Sarah J. Moore, Raphael N’Guessan

**Affiliations:** 1 Centre de Recherche Entomologique de Cotonou, Cotonou, Benin; 2 Environmental Health and Ecological Sciences Thematic Group, Ifakara Health Institute, Bagamoyo Research and Training Centre, Bagamoyo, Tanzania; 3 Swiss Tropical and Public Health Institute, Socinstr., 574051 Basel, Switzerland; 4 University of Basel, Petersplatz 1, 4003 Basel, Switzerland; 5 London School of Hygiene and Tropical Medicine, Keppel Street, WC1E 7HT London, UK

**Keywords:** Experimental hut, Mosquito, Long-lasting insecticidal net, Mosquito coil, West African hut, Ifakara hut, Benin

## Abstract

**Background:**

West African and Ifakara experimental huts are used to evaluate indoor mosquito control interventions, including spatial repellents and insecticides. The two hut types differ in size and design, so a side-by-side comparison was performed to investigate the performance of indoor interventions in the two hut designs using standard entomological outcomes: relative indoor mosquito density (deterrence), exophily (induced exit), blood-feeding and mortality of mosquitoes.

**Methods:**

Metofluthrin mosquito coils (0.00625% and 0.0097%) and Olyset® Net *vs* control nets (untreated, deliberately holed net) were evaluated against pyrethroid-resistant *Culex quinquefasciatus* in Benin. Four experimental huts were used: two West African hut designs and two Ifakara hut designs. Treatments were rotated among the huts every four nights until each treatment was tested in each hut 52 times. Volunteers rotated between huts nightly.

**Results:**

The Ifakara huts caught a median of 37 *Culex quinquefasciatus*/ night, while the West African huts captured a median of 8/ night (rate ratio 3.37, 95% CI: 2.30–4.94, *P* < 0.0001) and this difference in mosquito entry was similar for Olyset® Net and more pronounced for spatial repellents. Exophily was greater in the Ifakara huts with > 4-fold higher mosquito exit relative to the West African huts (odds ratio 4.18, 95% CI: 3.18–5.51, *P* < 0.0001), regardless of treatment. While blood-feeding rates were significantly higher in the West African huts, mortality appeared significantly lower for all treatments.

**Conclusions:**

The Ifakara hut captured more *Cx. quinquefasciatus* that could more easily exit into windows and eave traps after failing to blood-feed, compared to the West African hut. The higher mortality rates recorded in the Ifakara huts could be attributable to the greater proportions of *Culex* mosquitoes exiting and probably dying from starvation, relative to the situation in the West African huts.

## Background

During the Global Malaria Eradication Campaign (1955–1969) the need to evaluate the efficacy of residual insecticides such as DDT and Dieldrin for malaria campaigns motivated the development of experimental huts. The so-called West African experimental hut also referred to as “verandah trap hut” first built in Burkina Faso is a modified version of the huts designed by Smith & Hudson [1]. The design is based upon that of modern brick-walled houses with corrugated iron roofs. This type of experimental hut is included in WHOPES (World Health Organization Pesticide Evaluation Scheme) guidelines mainly because it is based on the design of local houses in Burkina Faso, simple and cheap to construct but also because it requires simple entomological collection methods [2]. Mosquitoes enter these huts through small window slits located on three sides and are trapped within the huts or in a largely walled verandah projected from the fourth side of the huts. There is a curtain between the room and the verandah that is usually raised during the night and lowered in the morning to prevent mosquitoes from flying back into the room. The hut allowed the assessment and comparison of several products against free flying malaria vectors and the West African design is now found in several African countries, including Burkina Faso, Benin, Cameroon and even Asia (Vietnam).

The Ifakara hut design [3] is a modified version of the “Portable hut” designed in Belize, Central America [4] that has similar dimensions to Tanzanian homes and includes features of East African hut designs including baffles to prevent mosquito egress [5], window traps [6] but with eave traps rather than verandah traps to capture mosquitoes that exit through eave gaps [7]. Both the West African and Ifakara huts are used to evaluate in WHO phase II trials the efficacy of mosquito control interventions, including insecticide [8–10] and repellent-based products [11, 12]. Though these huts have been in use for many years, only one study has determined the difference in LN efficacy that occur due to hut structure. This study was performed in Tanzania and compared the performance of Olyset® Net LN in three huts types, East African huts, Ifakara huts and West African experimental huts [13]. The East African and Ifakara huts were shown to capture substantially more mosquitoes, compared to the West African design. However, due to the high mortality in control huts (> 40%) recorded in the Tanzanian trial, it was not possible to compare net efficacy between huts types. Therefore, the question of whether the efficacy of a candidate indoor intervention changes with experimental hut design remains unaddressed.

Because they differ in design, each hut style may have benefits or weaknesses depending on the characteristics of the intervention under evaluation. This study aimed to compare the efficacy of a standard insecticidal net (Olyset® Net) and a mosquito repellent coil (metofluthrin), both evaluated in each type of huts side-by-side in Benin.

## Methods

### Study site

The study was conducted at Donoukin, a suburban village on the outskirts of Porto Novo, the administrative capital of Benin. The site supports the breeding of *Anopheles gambiae* M form, mostly pyrethroid-resistant with a high frequency of *kdr* (> 90%) and increased activity of cytochrome P450s [14]. The nuisance mosquito *Culex quinquefasciatus* is present in abundance year round and shows resistance to pyrethroids, carbamates and organophosphates [15].

### Experimental huts

Two West African and two Ifakara huts were constructed approximately 50 m apart from one another in a row between mosquito breeding sites and human habitations to maximize interception of host-seeking mosquitoes. Huts of the same design were constructed in a row, and the West African huts were located behind the Ifakara experimental huts.

The West African experimental huts are 2.5 m long × 1.75 m wide × 2 m high. The walls are made of concrete bricks and the roof of corrugated iron. A plastic cover is stretched under the roofing as a ceiling to facilitate the catching of mosquitoes. Entry of mosquitoes occurs through four slits, 1 cm wide, located on three sides of the hut. These slits were designed to prevent mosquitoes from escaping once they entered the hut. Mosquitoes can egress into a large verandah trap projecting from the fourth side. Each hut stands on a concrete base surrounded by a water-filled moat to exclude scavengers such as ants and spiders.

The Ifakara experimental huts design are 6.5 m long × 3.5 m wide × 2 m high. The walls are made of canvas lined with reed matting with a corrugated iron roof and a 10 cm eave gap on all sides through which mosquitoes enter and are prevented from leaving by netting baffles. Mosquito entry points are interspersed with eight eave interception traps fitted to huts on each side to catch eave-exiting mosquitoes in addition to four window exit traps: two on the front and two on the back of the huts. The huts are suspended above ground using concrete pillars surrounded by a water-filled moat to keep predators away from mosquitoes.

### Interventions

Control represented untreated white 100-denier polyester multifilament net (Siam-Dutch Mosquito Netting Co., Bangkok, Thailand) deliberately holed with six holes (4 × 4 cm) cut along the sides to simulate a torn net.

Olyset® Net is a long-lasting insecticidal net (LLIN), made of knitted polyethylene thread with permethrin at 20 g/kg (2% *w*/w) incorporated in it (Sumitomo Co, Tokyo, Japan). Both the control net and the Olyset net were deliberately holed with 6 holes (4 × 4 cm) cut along the sides to simulate wear and tear as per WHO guidelines [16].

Two doses of metofluthrin mosquito coils were evaluated: 0.00625% and 0.0097% (SC Johnson and Sons, USA). These were tested in conjunction with untreated, deliberately holed nets. One coil was used per hut per night for each of the two doses and burned for about 8 h.

### Study procedure

The study was a partially randomised Latin Square design where the four treatments were rotated weekly between the four huts (two West African huts and the two Ifakara designs) and volunteer sleepers rotated on a nightly basis to compensate for any variation in individual attractiveness. The study was conducted over 20 weeks with eight initial weeks between November 16^th^, 2011 and January 14^th^, 2012 followed by a further 12 weeks between June 13^th^, 2012 to September 2^nd^, 2012. Each treatment spent 52 nights in each hut. Each intervention was tested in each hut an equal number of times, i.e. 52 nights.

Four volunteer sleepers from nearby villages were recruited on written informed consent to sleep in the huts from 20:00 to 05:00 h each night. The area is highly endemic for malaria; sleepers were screened and treated for malaria, although no one fell ill during the study. The coil was lit at 20:00 h and was kept at a distance of 1 m away from the sleeper. In the morning, each volunteer collected mosquitoes from the walls, floors, under bed nets, ceilings, verandahs, and traps of the hut using torches and aspirators. Mosquitoes were transported to the laboratory for identification to species and scored as alive or dead, blood-fed or unfed. Surviving mosquitoes were held in plastic cups supplied with 10% glucose solution. The temperature was maintained at 27 ± 2 °C and relative humidity at 75 ± 10%. Mortality was scored 24 h post holding.

### Outcomes

The impact of each treatment was assessed according to the following parameters: (i) deterrence: percentage reduction in the number of mosquitoes caught in treated hut relative to the number caught in the control hut; (ii) induced exophily: percentage of the mosquitoes collected from exit traps of treated hut relative to percentage caught in exit traps of control hut; (iii) blood-feeding rate: percentage of the mosquitoes collected that were blood-fed in experimental huts; (iv) induced mortality: percentage of dead mosquitoes in treated hut relative to percentage dead in control hut; and (v) personal protection: the proportional reduction in the number of blood-fed mosquitoes relative to blood-fed mosquitoes in the control group.

### Data analysis

Data were collected on paper forms and entered into an Excel data base. Data were analyzed using the R statistical software version 2.15.0 [17] with a significance level of 0.05 for rejecting the null hypothesis. All generalized linear mixed models (GLMMs) were conducted using the *lme4* package [18]. Count data were modelled using a generalised linear model with a poisson distribution and logit link with treatment position, sleeper and day treated as random effects; treatment and hut type were fixed effects and fitted with an interaction. Proportional data (mortality, exophily and blood-feeding) were analysed using a generalised linear model with a binomial distribution and a logit link with position, and day treated as random effects; treatment, hut type and sleeper were fixed effects with treatment and hut type fitted with an interaction. Several GLMMs were run for each outcome and the final model selected was that with the lowest Akaike’s information criterion (AIC). Also, residuals were plotted using a histogram, quantile quintile plots and comparison with fitted values to ensure appropriateness of model selection.

## Results

A total of 7655 *Cx. quinquefasciatus* females were collected in the huts, of which 6263 were caught in the Ifakara huts and 1392 in the West African hut designs. Only 381 and 35 *Anopheles* were collected in the Ifakara and West African huts, respectively. Given the low numbers collected meaningful comparisons were difficult and so only *Cx. quinquefasciatus* data were analyzed and reported.

### Mosquito density

Overall, the Ifakara huts captured considerably more *Cx. quiquefasciatus* than the West African huts, whether or not they contained a product (Tables 1 and 2). The median in the Ifakara hut with control untreated net was 37, and the inter quartile range (IQR) 27–51 *Culex* mosquitoes per hut per night compared to 8 (IQR 4.0–14.5) in the West African huts (*P* < 0.001). The same trend was observed with Olyset nets, with a median of 7.5 (IQR 3.0–17.5) captured in the West African huts compared to a median of 29.5 (IQR 18.5–37.5) *Culex* mosquitoes in the Ifakara huts (*P* < 0.001). Deterrence with the low and high spatial repellent metofluthrin dosages was greater in the West African huts (78 and 79%) than in the Ifakara huts (28 and 49%), and a dose-dependent difference in mosquito entry was measurable in the Ifakara huts (Table 2).

**Table 1:** Deterrence of *Cx. quinquefasciatus* induced by Olyset® Net, metofluthrin coils at 0.00625% and 0.0097% and untreated control net in Ifakara and West African experimental huts

	No. caught	Median IQR caught	IRR	95% CI	*P*-value	*Z*	Deterrence %
Ifakara huts							
Control net	2067	37 (27–51)	1				–
Metofluthrin 0.00625%	1489	23 (15–36)	0.61	0.56–0.66	< 0.0001	-12.38	28
Metofluthrin 0.0097%	1054	21 (11–29)	0.65	0.59–0.71	< 0.0001	-9.55	49
Olyset® Net	1658	29.5 (18.5–37.5)	0.82	0.75–0.90	< 0.0001	-4.02	20
West African huts							
Control net	587	8 (4–14.5)	1				–
Metofluthrin 0.00625%	122	1 (0–4)	0.25	0.20–0.30	< 0.0001	-13.89	79
Metofluthrin 0.0097%	130	1 (0–4)	0.24	0.14–0.21	< 0.0001	-17.75	78
Olyset® Net	553	7.5 (3–17.5)	0.84	0.73–0.96	0.01	-2.48	6

**Table 2:** Measurements of deterrence compared between Ifakara and West African experimental huts for control net, Olyset® Net and metofluthrin coils at 0.00625% and 0.0097%

	No. caught	Median IQR caught	RR	95% CI	*P*-value	*Z*	Deterrence %
Control net							
West African hut	587	8 (4–14.5)	1				–
Ifakara hut	2067	37 (27–51)	3.37	2.30–4.94	< 0.0001	6.23	–
Olyset® Net							
West African hut	553	7.5 (3–17.5)	1				6
Ifakara hut	1658	29.5 (18.5–37.5)	3.29	2.24–4.84	< 0.0001	6.08	20
Metofluthrin 0.00625%							
West African hut	122	1 (0–4)	1				78
Ifakara hut	1489	23 (15–36)	8.58	5.68–12.96	< 0.0001	10.21	28
Metofluthrin 0.0097%							
West African hut	130	1 (0–4)	1				79
Ifakara hut	1054	21 (11–29)	12.69	8.40–19.15	< 0.0001	12.09	49

### Induced exophily

Results in Table 3 show that the exophily associated with Olyset nets relative to the control in Ifakara huts (OR: 4.44, 95% CI: 3.48–5.66, *P* < 0.001) was similar to that of West African huts (OR: 3.91, 95% CI: 2.84–5.38, *P* < 0.001). *Cx quinquefasciatus* mosquito that exit from the Ifakara huts was approximately double that of West African huts (64 *vs* 35%) in the control, odds ratio (OR) 4.18 (95% CI: 3.18–5.51, *P* < 0.001) (Table 4). This trend was similar across all treatments with consistently greater egress with each treatment from the Ifakara huts than from the West African huts (Table 4).

**Table 3:** Exopily induced by Olyset® Net, metofluthrin coils at 0.00625% and 0.0097% and untreated control net in Ifakara huts and West African experimental huts

	No. caught	Exiting % (95%CI)	Median IQR exiting	OR	95% CI	*P*-value	*Z*
Ifakara huts							
Control net	2067	64 (62–66)	23 (14–33.5)	1			
Metofluthrin 0.00625%	1489	82 (80–84)	20 (13–28)	2.76	2.28–3.35	< 0.0001	10.33
Metofluthrin 0.0097%	1054	85 (83–87)	18 (10–24)	3.22	2.55–4.07	< 0.0001	9.84
Olyset® Net	1658	88 (86–89)	27.5 (17.5–34.5)	4.44	3.48–5.66	< 0.0001	12.02
West African huts							
Control net	587	35 (31–39)	2 (0.5–3)	1			
Metofluthrin 0.00625%	122	49 (40–56)	0 (0–2)	2.14	1.37–3.34	< 0.0001	3.33
Metofluthrin 0.0097%	130	40 (31–48)	0 (0–1)	1.52	0.98–2.36	0.06	1.87
Olyset® Net	553	60 (56–64)	4 (2–11)	3.91	2.84–5.38	< 0.0001	8.36

**Table 4:** Measurements of induced exophily compared between Ifakara and West African experimental huts for control net, Olyset® Net and metofluthrin coils at 0.00625% and 0.0097%

Exophily	No. caught	Exiting % (95% CI)	Median IQR exiting	OR	95% CI	*P*-value	*Z*
Control net							
West African hut	587	35 (31–39)	2 (0.5–3)	1			
Ifakara hut	2067	64 (62–66)	23 (14–33.5)	4.18	3.18–5.51	< 0.0001	10.18
Olyset® Net							
West African hut	553	60 (56–64)	4 (2–11)	1			
Ifakara hut	1658	88 (86–89)	27.5 (17.5–34.5)	4.77	3.56–6.37	< 0.0001	10.53
Metofluthrin 0.00625%							
West African hut	122	49 (40–56)	0 (0–2)	1			
Ifakara hut	1489	82 (80–84)	20 (13–28)	5.41	3.54–8.28	< 0.0001	7.79
Metofluthrin 0.0097%							
West African hut	130	40 (31–48)	0 (0–1)	1			
Ifakara hut	1054	85 (83–87)	18 (10–24)	8.88	5.74–13.73	< 0.0001	9.82

### Blood-feeding rate and personal protection

The results of blood-feeding rates recorded by both hut designs using the different treatments are presented in Tables 5 and 6. There were significantly lower blood-feeding rates of *Culex* mosquitoes in Ifakara huts with control nets (25%) than in the West African huts (56%) (OR: 0.20, 95% CI: 0.15–0.28, *P* < 0.001). In both type of huts personal protection was highest with Olyset nets, measured at 96% in Ifakara huts and 93% in West African huts although this was statistically different between hut types: (OR: 0.29, 95% CI: 0.15–0.57, *Z* = -3.57, *P* < 0.001) (Table 6). The experiment demonstrated a dose-dependent effect on personal protection induced by the metofluthrin coils in the Ifakara huts (62% protection for low dose and 81% protection for high dose) (Table 6). However, the dose effect was not seen in the West African huts: 87 *vs* 89% personal protection with the lower and higher dose, respectively. The overlapping of the confidence intervals for % blood-feeding suggests that the West African huts did not discriminate a dose effect.

**Table 5:** Blood-feeding rates of pyrethroid resistant *Culex quinquefasciatus* in Ifakara and West African experimental huts

	No. caught	Total blood-fed	Median IQR blood-fed	OR	95% CI	*P*-value	*Z*	Blood-feeding % (95% CI)	Personal protection (%)
Ifakara huts									
Control net	2067	516	8.5 (3.5–12)	1				25 (23–27)	–
Metofluthrin 0.00625%	1489	194	2 (0–5)	0.36	0.28–0.46	< 0.0001	-8.16	13 (11–15)	62
Metofluthrin 0.0097%	1054	95	1 (0–3)	0.33	0.25–0.44	< 0.0001	-7.63	9 (7–11)	81
Olyset® Net	1658	17	0 (0–0)	0.03	0.02–0.05	< 0.0001	-13.76	1 (0.5–1.5)	96
West African huts									
Control net	587	329	4.5 (1.5–8.5)	1				56 (52–60)	–
Metofluthrin 0.00625%	122	41	0 (0–1)	0.33	0.20–0.54	< 0.0001	-4.38	34 (26–42)	87
Metofluthrin 0.0097%	130	35	0 (0–1)	0.19	0.11–0.31	< 0.0001	-6.72	27 (19–35)	89
Olyset® Net	553	22	0 (0–0)	0.02	0.01–0.03	< 0.0001	-14.24	4 (2–6)	93

**Table 6:** Measurements of blood-feeding rates compared between Ifakara huts and West African experimental huts for control net, Olyset® Net and metofluthrin coils at 0.00625% and 0.0097%

	No. caught	Total blood-fed	Median IQR blood-fed	OR	95% CI	*P*-value	*Z*	Blood-feeding % (95%CI)	Personal protection %
Control									
West African hut	587	329	4.5 (1.5–8.5)	1				56 (52–60)	–
Ifakara hut	2067	516	8.5 (3.5–12)	0.20	0.15–0.28	< 0.0001	-10.10	25 (23–27)	–
Olyset® Net									
West African hut	553	22	0 (0–0)	1				4 (2–6)	93
Ifakara hut	1658	17	0 (0–0)	0.29	0.15–0.57	< 0.0001	-3.57	1 (0.5–1.5)	96
Metofluthrin 0.00625%									
West African hut	122	41	0 (0–1)	1				34 (26–42)	87
Ifakara hut	1489	194	2 (0–5)	0.22	0.13–0.36	< 0.0001	-5.96	13 (11–15)	62
Metofluthrin 0.0097%									
West African hut	130	35	0 (0–1)	1				27 (19–35)	89
Ifakara hut	1054	95	1 (0–3)	0.36	0.22–0.60	< 0.0001	-3.92	9 (7–11)	81

### Induced mortality

Mosquito mortality induced by all treatments in the Ifakara huts was consistently higher than in the West African huts (Tables 7 and 8). This was particularly pronounced with the high dose metofluthrin coil where mortality was 29% (95% CI: 26–32) in the Ifakara huts compared to 3% (95% CI: 0.01–6) in the West African huts, odds ratio 13.93 (95% CI: 4.90–39.63, *P* < 0.001). Also, mortality in the Ifakara huts with Olyset nets (25%) (95% CI: 2–27) was higher than in the West African huts (10%) (95% CI: 7–12), odds ratio 3.95 (95% CI: 2.55–6.12, *P* < 0.001). Control mortality was also marginally higher in the Ifakara huts (9%) (95% CI: 8–10) than the West African huts (6%) (95% CI: 4–8); the difference though significant was borderline, odds ratio 1.64 (95% CI: 1.01–2.66, *P* = 0.04) and both values were within acceptable levels as recommended by WHOPES (< 10%).

**Table 7:** Mortality induced by Olyset® Net, metofluthrin coils at 0.00625% and 0.0097% and untreated control net in Ifakara and West African experimental huts

	No. caught	Total dead	Median IQR dead	OR	95% CI	*P*-value	*Z*	Mortality % (95% CI)
Ifakara huts								
Control net	2067	195	2 (0–5.5)	1				9 (8–10)
Metofluthrin 0.00625%	1489	204	3 (0–6)	1.71	1.33–2.20	< 0.0001	4.14	14 (12–16)
Metofluthrin 0.0097%	1054	307	5 (1–10)	4.46	3.44–41.83	< 0.0001	11.29	29 (26–32)
Olyset Net®	1658	423	7 (5–11)	4.12	3.13–5.78	< 0.0001	10.09	25 (2–27)
West African huts								
Control net	587	35	0 (0–0.5)	1				6 (4–8)
Metofluthrin 0.00625%	122	12	0 (0–0)	1.49	0.71–3.12	0.29	1.05	10 (5–15)
Metofluthrin 0.0097%	130	4	0 (0–0)	0.52	0.18–1.53	0.23	-1.19	3 (0.1–6)
Olyset Net®	553	53	0 (0–1)	1.69	1.01–2.84	0.05	1.99	10 (7–12)

**Table 8:** Measurements of mortality compared between Ifakara and West African experimental huts for control net, Olyset® Net and metofluthrin coils at 0.00625% and 0.0097%

Mortality	No. caught	Total dead	Median IQR dead	OR	95% CI	*P*-value	*Z*	Mortality % (95%CI)
Control								
West African hut	587	35	0 (0–0.5)	1				6 (4–8)
Ifakara hut	2067	195	2 (0–5.5)	1.64	1.01–2.66	0.04	2.01	9 (8–10)
Olyset® Net								
West African hut	553	53	0 (0–1)	1				10 (7–12)
Ifakara hut	1658	423	7 (5–11)	3.95	2.55–6.12	< 0.0001	6.14	25 (2–27)
Metofluthrin 0.00625%								
West African hut	122	12	0 (0–0)	1				10 (5–15)
Ifakara hut	1489	204	3 (0–6)	1.85	0.92–3.72	0.08	1.71	14 (12–16)
Metofluthrin 0.0097%								
West African hut	130	4	0 (0–0)	1				3 (0.1–6)
Ifakara hut	1054	307	5 (1–10)	13.93	4.90–39.63	< 0.0001	4.94	29 (26–32)

## Discussion

The data suggest that the design of each hut type impacts vector behaviour differently. The Ifakara hut with control net was almost four-fold more attractive to *Cx quinquefasciatus* than the West African huts, owing to access of mosquitoes to the hut through the eave gaps (between 50 and 60 cm wide each) on all four sides compared to entrance *via* the small window slits (1 cm wide each) on three sides of the West African huts.

The analysis of the exophily rates in the Ifakara huts showed that when mosquitoes enter huts naturally for a blood meal source and sleepers in those huts are protected by untreated bednet, a large proportion of hungry females (in the order of 64% in the present trial) exit the huts. The egress from these huts was further increased by the presence of Olyset net or metofluthrin repellent interventions, with induced exophily reaching 88% for the Olyset net in the Ifakara huts and 60% in the West African huts. Against *An. arabiensis* in Tanzania, Olyset net in the Ifakara huts also induced similar exophily rate of 90% [9] although lower deterrence was observed in the Tanzanian field study than in the study here reported. An explanation could be that a larger number of experimental huts were used in the Tanzanian study over a longer duration that may have reduced the impact of spatial heterogeneity on relative mosquito densities. The lower deterrence in Tanzania could also be due to species differences; *Cx. quinquefasciatus* in the current Beninese trial versus *An. arabiensis* in Tanzania. Studies have shown that *An. arabiensis* is likely to exit huts when a blood meal source is protected by a bednet [9]. The present study shows a similar trend for *Cx. quinquefasciatus* with only blood-fed mosquitoes remaining inside the Ifakara huts. The consequence is that the proportions of mosquitoes remaining inside and killed in the Ifakara huts were small (25% of resistant *Culex* with Olyset net), a rate higher than that killed by Olyset net in Tanzania (4% *Culex* mortality) in same hut type [9]. In the West African hut, Olyset net killed only 10% of *Culex* mosquitoes, a rate similar to previous trials conducted in the same study area [8]. The proportion of *Culex* mosquitoes dying in the presence of Olyset net was higher in the Ifakara hut than in the West African hut. This finding is at odds with a similar study comparing Olyset net in a side by side comparison of the Ifakara and the West African huts in Tanzania where mortality did not differ [18]. However, control mortality in this study was unacceptably high (41%) so the insecticide induced mortality could not be detected, *vs* < 10% in our current study, excluding any comparison between the two studies. Moreover, the performance of the three experimental huts tested in the Tanzanian study was assessed on a pooled set of mosquitoes [*An. gambiae* (*s.l*.), *An. funestus*, *Mansonia* and *Culex* mosquitoes] rather than presenting the data by species, again making the comparison of mortality measurements between the two studies impossible.

In the current study, the significant difference in mortality observed when Olyset® Net was tested in the Ifakara hut (25%) *vs* in the West African hut (10%) is a result of the difference in hut structure. First, the roof of the Ifakara hut is surrounded by eaves and has four window exit traps, which increased the exit rate of mosquitoes after an unsuccessful attempt to blood-feed. This is supported by our data which showed greater exophily rate with this hut type. The mosquitoes are subsequently trapped in the exit space where they likely died from starvation. This might account for the higher mortality with the Olyset net in the Ifakara hut. In future studies, it may be useful to provide mosquitoes with a sugar solution to prevent starvation. This difference in hut structure where mosquitoes are retained in the West African huts until morning may have impacted blood-feeding estimate with mosquitoes more likely to make multiple attempts to blood-feeding rather than exiting once they fail to feed. This would seem unrealistic, particularly within those rural households that have windows and eaves that allow mosquitoes to freely enter and exit, and consequently may not reflect the natural expression of the mosquitoes behaviour, especially where users are protected with nets.

The usual egress rates of *Culex* mosquitoes as estimated in the West African hut in the absence of interventions from several trials averaged 30% [19–22] compared to 64% in the current study and over 90% in the previous study [9]. It is understandable that a good rationale behind the West African design is fixing the hut tight that is also based on the style of houses used in Burkina Faso to allow a more accurate estimation of the denominator in measuring the different entomological parameters, e.g. the total number of mosquitoes that initially entered that hut. However, the Ifakara huts have eave gaps and prevent egress of mosquitoes *via* the eaves using baffles [2], so the mosquitoes are retained within the huts or captured in eave and window exit traps.

The West African huts could be easily amended to accommodate natural exit route of mosquitoes that best reflects reality. For example, one could overcome mosquito restriction within huts by concrete-sealing the verandah compartment and funnelling slits on the top wall of that verandah compartment for the mosquitoes to exit the huts freely but limit their return. A small door designed onto the meshed area projected on the back of that verandah will allow morning collection and a good estimate of mosquitoes that would normally have escaped the huts.

While deterrence induced by the repellent interventions (coils) was apparent in both hut styles the effect was more pronounced in the West African hut design (78–79%) compared to the effect observed in Ifakara huts (28–49%). Since mosquitoes are forced to enter the huts through narrow slits, it is likely that the concentration of volatile pyrethroid encountered will be far higher than in the larger and more open Ifakara huts. The airflow of an experimental hut is an important consideration when considering vapour acting interventions, and the Ifakara huts were shown to have similar indoor air movements as local houses [12]. Also, when considering the impact of indoor residual spray (IRS), it is important to consider the temperature and airflow inside of experimental huts as this will lead to volatilization of insecticide particles [23]. Unfortunately, comparison of temperature and wind movement between huts used in this study was not made.

This study showed differences in huts feature between West and Ifakara huts; however, both types are equivalently suitable to evaluate products of any kind, i.e. whether insecticidal or repellent. WHO recommends that a novel product should be assessed alongside a positive control of the same family products against the same vector species at a given site and type of hut; it should always be possible to evaluate the relative efficacy of a product in a given type of hut despite the structural difference in feature.

## Conclusions

The data show clear differences between parameters measured by each hut with each parameter being consistently differently measured for the control, Olyset® Net and spatial repellents. Of particular importance is the fact that the Ifakara huts caught a significantly higher number of *Cx quinquefasciatus* mosquitoes and allowed free exit of mosquitoes from huts when they do not obtain a blood meal whereas fewer mosquitoes can enter or leave the West African huts. This is an important consideration when evaluating interventions that require mosquito behaviour to be as normal as possible.

## Abbreviations

AIC: Akaike’s information criterion
CI: Confidence interval
GLMMs: Generalized linear mixed models
IQR: Interquartile range
IRS: Indoor residual spray
kdr: Knockdown resistance
LLIN: Long-lasting insecticidal nets
OR: Odds ratio
WHO: World Health Organisation
WHOPES: World Health Organisation Pesticide Evaluation Scheme
